# Sustainable formulation polymers for home, beauty and personal care: challenges and opportunities

**DOI:** 10.1039/d3sc04488b

**Published:** 2023-11-02

**Authors:** Christina A. R. Picken, Orla Buensoz, Paul D. Price, Christopher Fidge, Laurie Points, Michael P. Shaver

**Affiliations:** a Department of Materials, Henry Royce Institute, The University of Manchester Manchester M13 9PL UK michael.shaver@manchester.ac.uk; b Unilever R&D, Port Sunlight Laboratory Quarry Road East, Bebington, Wirral CH63 3JW UK

## Abstract

As society moves towards a net-zero future, the need to adopt more sustainable polymers is well understood, and as well as plastics, less visible formulation polymers should also be included within this shift. As researchers, industries and consumers move towards more sustainable products there is a clear need to define what sustainability means in fast moving consumer goods and how it can be considered at the design stage. In this perspective key challenges in achieving sustainable formulation polymers are highlighted, and opportunities to overcome them are presented.

## Abstract 2

As researchers, industries and consumers move towards more sustainable products there is a clear need to define what sustainability means in fast moving consumer goods and how it can be considered at the design stage.

## Graphical abstract

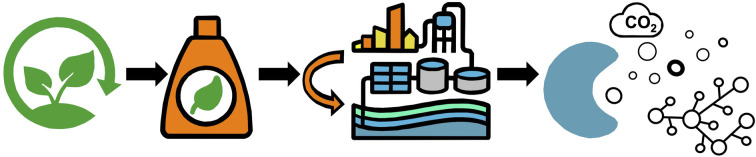

## Introduction

Synthetic polymers are ubiquitous in the modern world and permeate every aspect of our lives, owing to their inimitable versatility coupled with their low-cost and ease of synthesis from fossil-fuel based sources. While applications like plastic packaging are obvious in their appearance and form, the polymeric ingredients included in many fast-moving consumer goods (FMCGs), such as soft drinks, toilet paper, shampoos, over-the-counter medicines and washing detergents are often “invisible”. These products are low cost, high volume and account for a significant proportion of all consumer spending.^1^ Formulation polymers are key to the delivery of technical performance of many FMCGs. Polymeric additives perform many tasks, such as being used to alter texture and viscosity, increase stability and provide application-specific activity (*e.g.* polymeric surfactants) to different products which enables tailoring of the product to its use.^2,3^

The ingredients used in consumer goods are carefully assessed to ensure safety for both humans and the environment however, most are discarded into waste-water streams and some may be poorly biodegradable in the environment. There is therefore both the requirement and opportunity to ensure that ingredients are selected to be as sustainable as possible, reducing the planetary impact of doing business and ultimately helping to regenerate natural systems. Consideration of sustainability is multi-faceted, with factors such as greenhouse gas footprint, land use, water use and end-of-life fate all being important. Polymers typically used in FMCGs face two interconnected challenges: they are often derived from fossil fuels and do not necessarily degrade to biorelevant products at the end of the product lifecycle. Despite making up typically less than 10% by weight of FMCG products, the production of formulation polymers is estimated to be a sizeable 29–36 million tonnes per year.^4^

In this perspective we aim to provide insight into the importance of different polymer types, highlight some of the challenges in achieving sustainable formulation polymers, and identify areas of opportunity. Lastly, we make suggestions for future changes to help guide us towards success and avoid unintended consequences.

This article considers formulation polymers used in the context of FMCGs for beauty and personal care, and home care, and herein the term “formulation polymer” refers to these classes of polymer. For the purposes of this review, both agricultural and food polymers were not considered owing to their different use phases including end-of-life, lack of direct consumer contact and/or different regulatory requirements.

## Status quo of formulation polymers

Formulation polymers can be categorised as synthetic (*i.e.*, made from petrochemical feedstocks using chemical processes), biopolymers (*i.e.*, a polymer found in nature), semi-synthetic (chemically modified biopolymers) or bio-based (*i.e.*, made from natural feedstocks but still using chemical production processes). Most industrially relevant formulation polymers are synthetic and require petroleum-derived starting materials. The extraction and processing of fossil fuels is energy intensive and contributes significant greenhouse gas emissions, loss of biodiversity and decreases water security.^5^

Formulation polymers in home, beauty and personal care products ultimately end up being dissipated by dilution in wastewater streams following product use by the consumer. For products sold in multiple regions around the world, variations in wastewater treatment infrastructure mean that the path polymers take, and their ultimate destination, varies. In countries with developed wastewater infrastructure and sewage treatment plants, many polymers will end up adsorbed on sewage sludge, which may then be burned for energy recovery or spread on fields as fertiliser. In countries with less developed infrastructure the polymers will remain in water streams, ultimately being discharged into rivers and the marine environment. These different eventual fates mean that polymers are exposed to variations in factors such as water level, pH, temperature, ionic strength, redox conditions, and microbial population and load at their end-of-life.

Despite the minimal toxicological effects of current formulation polymers, they are generally slow to biodegrade, driven by their large molecular size, poor bioavailability and, in many cases, lack of functional handles in the carbon–carbon backbone. Biodegradation of formulation ingredients are assessed by standardised tests, for example in soil (*e.g.* OECD 304A), fresh water (*e.g.* OECD 301, OECD 302) and marine environments (*e.g.* OECD 306). However, these methods cannot account for all variables present in the natural environment and are thus conservatively designed to account for this variation. The tests work by monitoring the complete carbonisation of chemicals into CO_2_, inorganic minerals, H_2_O, and new biomass. Although these methods provide an indication of the propensity of an ingredient to biodegrade, they have limitations.^6^ Most tests were developed for small molecules and so the defined endpoint of complete biodegradation may not account for the ecotoxicological impact of oligomeric and molecular intermediates. Standards designed to measure the environmental biodegradation of polymers are also flawed as they lack uniformity and fail to account for the large number of environmental variables present.^7^

**Fig. 1:**
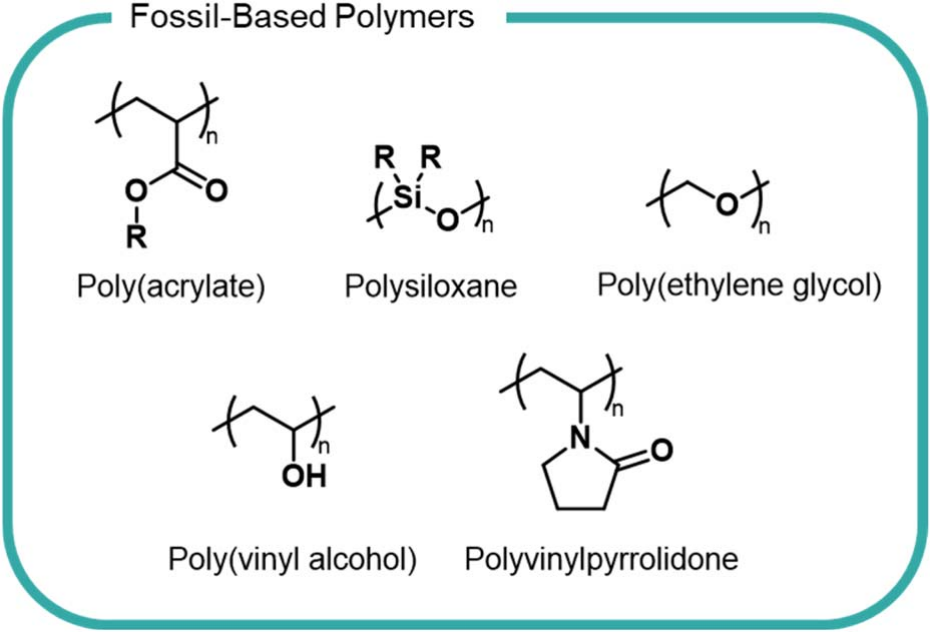
Structures of common current polymers used in home, beauty and personal care formulations.

There is a myriad of different formulation polymers, with diverse structures and properties,^8,9^ however they are based on a relatively small number of monomers and backbones (Fig. 1).^4^ This chemical convergency allows for similar processing and production methods, thereby reducing costs and emissions.

### Polymer structures

Polyacrylates, such as poly(acrylic acid) and polyacrylamide, are some of the most widely used formulation polymers and are synthesised *via* a radical polymerisation.^10^ The polymer properties are varied to suit specific applications. For instance, molecular weight is altered by addition of a chain-transfer agent which shortens polymer chains by reacting with the end-chain radical to interrupt growth. Topology is controlled by addition of a co-monomer with more than two polymerizable functional groups to give branched or cross-linked structures. The pendant carboxyl groups present in acrylic polymers (Fig. 1) lead to excellent water solubility owing to their ability to interact with H_2_O molecules. Acrylic polymers act as rheology modifiers by binding or immobilising water in liquid-based formulations to give products their desired flow behaviour. These polymers are also used as emulsifying agents and work by reducing surface tension between hydrophilic and hydrophobic ingredients to form a consistent texture. Acrylic polymers lack any useful chemical handles within the backbone to allow biodegradation to occur and are non-biodegradable according to OECD standards. In certain instances, it is possible to source acrylic monomers from bio-derived sources, which may reduce the environmental impacts of this polymer class, however the economic feasibility and overall life-cycle analysis is yet to be explored.^11,12^

Polysiloxanes (Fig. 1) are comprised of an alternating silicon and oxygen backbone with pendant functional groups (typically alkyl groups) substituted on the silicon atoms. These polymers are relatively inert as a consequence of free rotation of the polymer chain which results in optimal orientation of side chains creating a low energy surface. The strong Si–O and Si–C bonds also contribute to a lack of reactivity and consequent lack of degradability.^13^ Linear and cyclic polysiloxanes are an important class of compounds in home and personal care products, finding use as lubricating agents and anti-foaming agents.^14–16^ They have widely been considered to have low toxicity towards living organisms and humans, as evidenced by experimental studies showing minimal or no toxicological effects.^17,18^ However, recent studies have brought into question the accuracy and consistency of these studies, suggesting that we cannot generalise commercial polysiloxanes as one polymer and must consider all chemical species present when considering toxicological effects.^19^ Polysiloxanes are typically removed from wastewater through absorption onto sewage sludge, which may then be applied to agricultural fields and therefore released into the environment.^20^ Polydimethylsiloxane (PDMS) is the simplest polysiloxane and can abiotically degrade in soils containing Lewis acidic minerals (*e.g.* minerals with Fe^3+^ and Al^3+^ sites).^21^ However, it is unlikely PDMS degrades in sewage sludge as studies have shown the polymer fails to biodegrade under aerobic or anaerobic conditions in simulated environments.^20^

Poly(vinyl alcohol) (PVA) and polyvinylpyrrolidone (PVP) (Fig. 1) are two vinyl polymers widely used in beauty and home care products. PVA is a synthetic water-soluble polymer and is synthesised by the hydrolysis of poly(vinyl acetate), as the direct monomer, vinyl alcohol, is not possible to isolate due to its immediate conversion to the tautomeric acetaldehyde.^22^ PVA is used as the film material in liquid detergent capsules and as a binder and thickener in beauty and personal care products.^1^ The 1,3-diol moiety present in the PVA backbone makes it one of the only carbon backbone polymers that is susceptible to biodegradation.^22–24^ The metabolic pathway of PVA degradation has two distinct steps, requiring two different enzymes. Firstly, hydroxyl groups are converted into a β-diketones by a secondary alcohol oxidase enzyme before hydrolytic cleavage of the C–C bonds between two carbonyls by a β-diketone hydrolase enzyme.^24,25^ However, PVA biodegradation is known to only occur in the presence of suitable bacteria. Studies utilising real-world sewage sludge have shown PVA degradation to be significantly reduced compared with laboratory degradation.^26^

PVP possesses a pendant lactam ring (Fig. 1) and is synthesised by radical polymerisation of *N*-vinyl-2-pyrrolidone. The lactam ring can hydrogen bond rendering PVP soluble in water and many organic solvents, which facilitates its use across home and personal care industries as a film-former and thickening agent. The polymer is non-toxic and bio-compatible. For example, PVP fed to experimental animals was shown to be simply excreted with no indication of accumulation.^27^ The biological inertness of PVP results in an almost complete lack of degradability, as evidenced by biodegradation studies. The ubiquity and lack of degradability of PVP has led to its detection in wastewater streams.^28^ PVP has been shown to complex well onto sewage sludge however few studies have investigated its true environmental fate.^29^

PEG poly(ethylene glycol) (Fig. 1) is a key component of many personal care and beauty formulations. PEG is a water-soluble polyether, synthesised on an industrial scale by the ring-opening polymerisation (ROP) of ethylene oxide which, in turn, is typically made from fossil-derived ethylene (although bioderived streams are becoming increasingly available). PEG is available at varying molar masses, with different end groups, and in different co-polymers – each variation imbuing the polymers with different properties. PEG polymers act as an emulsifier in creams and lotions and as a wetting agent. Fatty-acid modified PEGs which contain both hydrophilic and hydrophobic groups are used as non-ionic surfactants in home-care formulations. Despite the relative stability of the polyether backbone, PEG can be biodegraded under aerobic and anaerobic conditions, relying on the free alcohol end-groups for an oxidative mechanism of degradation to occur.^30,31^

Biopolymers are abundant in nature and many have been repurposed as formulation ingredients, for instance starch has been used as thickening agents in cosmetics.^32^ Biopolymers such as polysaccharides possess different topologies and functionalities, including cationic, anionic, and amphoteric functional groups.^33^ The structures of biopolymers are limited in their raw form and chemical modifications are made to widen their scope of use as formulation polymers, to produce bio-based polymers. Modifications include acetylation, alkylation and halogenation, acidification by succinylation, and the formation of salts.^33^

The polymer backbone is the primary factor in determining polymer properties, however there are many additional factors at play. Understanding polymer–solution interactions is key to rational design of new formulation polymers. For instance, thickening can be achieved using crosslinked polymers by the entangled nature of polymer chains or by association interactions of hydrophobic domains.^32^ The choice of polymer class, molar mass, architecture, and inclusion of side-chain functional groups all contribute to tuning of macromolecular properties to suit a specific function. For formulation polymers, the main performance considerations are the behaviour in solution (or as a liquid), ability to stabilise a formulation and the ability to affect rheological behaviour. When developing new polymers it is imperative to factor in sustainability aspects at the very initial stages.^34^ Moreover, we must take a holistic view of polymer sustainability and understand that designing for biodegradability is just one part of the move towards a circular economy. Consideration of social and economic sustainability must be coupled with environmental sustainability in order to actualise the envisioned end-of-life fates of designed polymers.^35^

## Formulation polymer design challenges

**Fig. 2:**
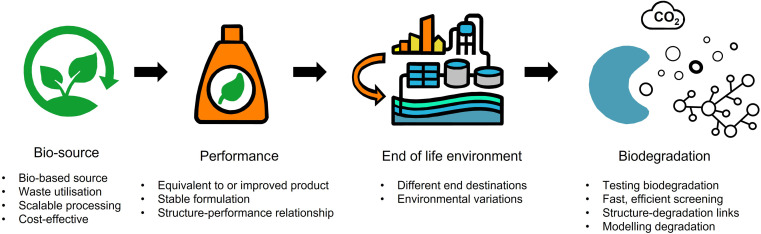
Summary of challenges and requirements in developing sustainable polymers for formulations.

The fundamental tenets of a circular economy are to (i) eliminate waste and pollution, (ii) ensure products and materials are retained at their highest value, and (iii) regenerate nature.^36^ For a formulation polymer, circularity can be achieved by decoupling from fossil-derived resources and moving towards recycled or waste feedstocks as well as ensuring polymers rapidly biodegrade.^9^ The scale of the challenge should not be understated. Herein, we explore the key challenges surrounding the development of formulation polymers for personal, beauty and home care products that are more safe and more sustainable by design (Fig. 2). The starting feature is performance. Polymers are used for a variety of functions and are designed to meet the requirements of their performance. For customers, the performance of a formulation is integral to the decision to (re)purchase and (re)use a product. The switch to more sustainable formulations must not risk reducing the quality and efficacy of the performance. Furthermore, less effective products in the name of sustainability may lead to countereffects. For example, a cleaning product that doesn't foam as well may result in more of the product being used to perform its function.

### Bio-sourcing

As stipulated by the circular economy framework, designing for circularity can utilise renewable resources (*i.e.* biomass waste, with a more circuitous route to circularity) or recycled polymer or chemical feedstocks (*i.e.* with a more tightly controlled but shorter path to circularity). The diverse fate of FMCG formulation polymers suggests designing for the biosphere may be preferential. Many natural polymers exist in nature such as polysaccharides and polypeptides, which are already used as formulation ingredients (starch, alginates, chitosan).^37,38^ Recently, investigations into biopolymers has expanded to include new sources (*e.g.* citrus skins, spent coffee grounds),^39–42^ new modifications (*e.g.* semi-synthetic lignin or building degradable links into non-degradable polymers).^43–45^ and new methods of extraction (*e.g.* by bacterial fermentation).^46–48^ In addition, natural feedstocks have been used to source monomers and reagents for the synthesis of bio-based polymers (*e.g.* poly(lactic acid) or poly(hydroxyalkanoates)).^9,49^

Bio-based polymers are not, by definition, more sustainable than fossil-derived polymers. Many bio-based polymer life cycle assessments (LCAs) highlight potential issues with farming and eutrophication risks as the main negative environmental impacts.^50,51^ It is predicted that without careful consideration, an increased demand in bio-based feedstocks increases demand on land use and therefore could negatively impact food production and biodiversity.^52,53^ To avoid harmful consequences and achieve true sustainability, the wider implications of changing feedstocks need to be considered.^54,55^ To this end, biomass produced as by-product from existing food and agriculture industries offers a beneficial and compatible opportunity.^53,56,57^

Furthermore, as described above, a variety of monomers, chemistries, structures and functionalities gives rise to a diverse catalogue of polymer functions used in a wide range of applications.^8,9^ Despite the functional variety, a majority of formulation polymers are derived from a limited number chemical feedstocks.^4^ Feedstock quality must be reliable and clean to ensure a consistent product is distributed to customers. Obtaining a pure polymer or monomer stream requires a degree of processing which itself needs to be scalable, efficient, affordable, and not place high demands on water security or resource usage. An opportunity exists to identify key bio-based resources which facilitate flexibility in the processing to provide a variety of structures for different functions.

### Economies of scale

For high volume commodity products such as FMCGs, cost of goods is typically low. Affordable and reliable polymer ingredients that meet performance and reproducibility demands are therefore needed. Economic analysis of solid plastic production found that bio-based feedstocks contribute to increased costs of two to three times compared to conventional fossil-derived feedstocks.^4^ Even when used in low percentages of a formulation, a significant increase in cost of degradable formulation polymer can drive up the cost of the product which can have a detrimental impact on affordability worldwide. In the short-term, as few sustainable alternatives are available on the market, this may significantly reduce uptake and limit scaling of more sustainable alternatives. To keep costs low a balance between cost and function is often finely tuned.

An option to affordably compete with current formulation polymers and build into a circular model is to base future polymer design on existing feedstocks or waste products which are already produced at scale. The extraction and processing of bio-based feedstocks requires fast, robust and efficient processes which are safe and scalable, utilising inexpensive reagents and catalysts, and low volumes of solvents. Streamlining adaptable processes to extract multiple products from one feedstock will be economically beneficial. The relatively small scale of formulation polymers when compared to the scale of plastics production creates a duality of pressures, where co-production with other petroleum products skews system economics. Utilising mass balance of renewable and more sustainable feedstocks may offer a route to scale new feedstocks whilst recouping increased costs and avoiding the large capex costs associated with new processes.

### Biodegradation

As previously discussed, polymers impart beneficial properties to formulations. A key challenge in designing biodegradable formulation polymers is achieving a product that is stable in formulations (across potentially lengthy supply chains from production to consumer use) but is quickly and completely degradable once diluted at the end of the useful lifetime to reduce any potential impacts on water resources.^4,36^ The degradation characteristics (*i.e.* rate and extent of degradability) is a governing factor when designing new degradable bio-based polymers.

Biodegradable polymers are defined as polymers which break down to defined metabolic end points without pollution or deleterious effects in a reported time frame.^4^ Whilst current formulation polymers in use are assessed as safe, many are non-biodegradable and there is a growing desire from industry, consumers and government to move towards polymers which do not remain in the environment.^58–61^

Degradation can be promoted by biological means such as bacteria, fungi or enzymes, either aerobically or anaerobically, or by abiotic chemical processes *via* hydrolysis, oxidation or photolytic cleavage.^62^ The large size of polymers impairs their uptake by microbial cells.^63,64^ Microorganisms release enzymes which can degrade specific bonds and functional groups extracellularly, creating smaller molecules which can then be metabolised.^65^ In all ideal cases, the end products of biodegradation are carbon dioxide (and methane for anaerobic degradation), water, inorganic minerals and new biomass.^65^

The biodegradability of a formulation polymer is governed by two main features; the chemical and structural design of the polymer material and the conditions of the environment at end-of-life.^66^ Some natural biodegradable polymers such as polysaccharides and polypeptides have evolved with their ecosystems to include labile bonds within the polymer chain susceptible to enzymatic or chemical cleavage.^67^ Across different polymers, whether naturally occurring or not, the same class of cleavable bonds can have significantly different rates of degradation. Enzymatic and chemical cleavage can be affected by the polymer size, stereochemistry of the monomer units, the sequence of units and functional groups neighbouring the cleavable site, degree of crystallinity, and the chain end groups.^66^ The modification of polymer structures can therefore increase or decrease the rate of degradation which presents both a risk and an opportunity to fine tune degradation rates.

Besides polymer design, degradability is also dependent on the environment of release. Water-based formulations are typically washed down wastewater routes which vary worldwide. In the developed world, wastewater is largely treated in municipal water treatment sites in which it undergoes enzymatic and chemical degradation before entering water courses such as rivers and lakes. Regional and temporal differences in microbial populations and enzymes may alter the degradation rates.^68^ The ability of polymers to adsorb onto waste sludge changes residence time in a water treatment site which makes degradation rates difficult to predict.^8^ Furthermore, sewage sludge can end up in agricultural applications which increases the environmental reach of non-degradable polymers.^8,69^ Elsewhere in the world, direct discharge of products by consumers into rivers, lakes and seas could limit enzymatic degradation activity or function. Whilst the polymers are assessed as safe for the environment, their specific fates remain unknown. Variations across the two aqueous pathways include pH, temperature, ionic strength, redox conditions and microbial populations. With the combined differences in mind, it is necessary to consider the likely wastewater pathway when designing or choosing a biodegradable formulation polymer.

### Biodegradation testing

Degradation guidelines for small molecules and structural polymers are outlined by the OECD (as discussed earlier) and involve variations of incubation of the test sample in defined conditions to measure the carbon (CO_2_ or CH_4_) evolved or O_2_ taken up over time.^7^ Whilst accepted to be conservative and provide a strong indication of the biodegradation of a test item in the environment, the limitations with current degradation testing was summarised by Zumstein and coworkers,^66^ which included (i) not accounting for O_2_ sequestration mechanisms, (ii) a potential decrease in enzymatic activity by dilution of inoculation media, (iii) accounting for temperature variability where degradation rates do not vary linearly with temperature, (iv) extrapolation of time scales in largely variable systems and (v) tests were largely designed for small molecules and are not as well suited to large molecules such as polymers where issues with bioavailability needs to be overcome.

The cost and duration of the current suite of biodegradation tests mean that they are typically only applied to final ingredients, or to a small number of late-stage prototype materials in a development project. Ideally, biodegradation in multiple environmental compartments would be screened (experimentally or *in silico*) alongside other fundamental performance parameters early in the development process of new families of more sustainable formulation polymers. This would both enable decisions to be taken on prototype ingredients with more information, but also enable the development of a larger dataset of polymer biodegradability data, feeding general understanding of these processes and allowing the development of quantitative structure-biodegradability relationships. The development of new rapid experimental and computational capabilities in this area remains a key unmet need for the industry.^70^

### Environmental safety

Considering the number of formulations consumers use on regular basis, it is clear that the polymeric ingredients within them need to be safe for human and environmental contact. Formulation ingredients are risk assessed for eco-toxicological effects although most polymers have been categorised as benign owing to their large size which renders them non-bioavailable.^59^ As degradable polymers are broken down to oligomers and small molecules, the eco-toxicological profiles of the intermediates also need to be considered. Furthermore, the products and degradation products must be demonstrated to be safe to wastewater streams and environments which they will be exposed to. The potential biological systems that a formulation product could encounter – whether bacterial, fungal, plant or animal – is so broad that screening for all potential environmental conditions or ecological interactions is not feasible. In addition, the types of catalyst, reagents and by-products used in production processes for new formulation polymers will also need to be judiciously selected to reduce potential accumulation in the environment, especially if non-intentionally added substances could be produced from cross-reactivity. We must also recognise that biodegradation is not instantaneous, and the structures formed upon breakdown of future polymers are diverse (monomers, dimers, oligomers). These factors should inform future testing and modelling of environmental impacts.

## The new formulation polymer frontier

**Fig. 3:**
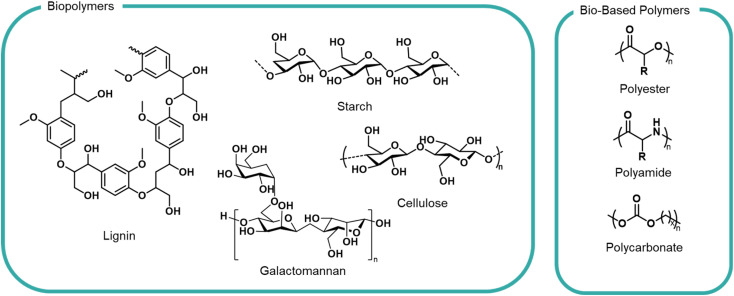
General structures of biopolymers and bio-based polymers investigated as formulation polymers.

Moving away from petrochemical feedstocks is a challenge for polymer industries for the reasons highlighted above. Fortunately, the natural world is abundant with a large variety of biopolymers from DNA to wood lignin to the webs of spiders – all of which possess known degradation and biological uptake pathways, *via* hydrolysis, bacterial or enzymatic means. This does not mean that all bio-based materials are appropriate feedstocks; many woody, waxy or tough biopolymers would not pass an OECD biodegradation test. We apply this sustainability lens to some notable families of polymers currently under development (Fig. 3).

### Next-generation degradable vinyl polymers

**Fig. 4:**
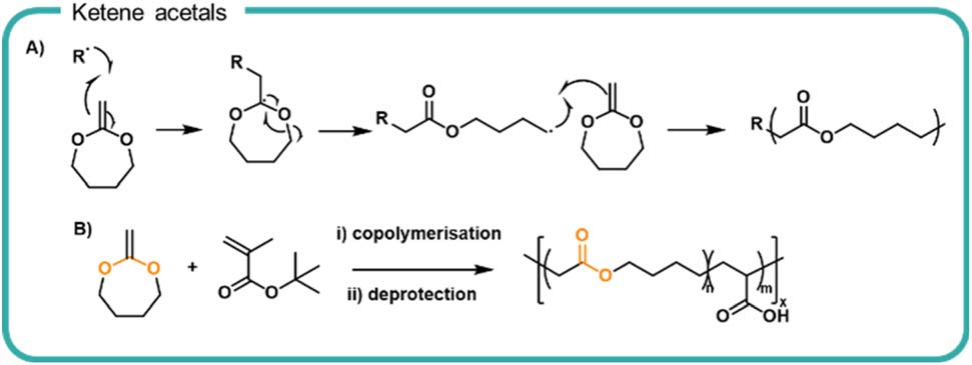
(A) Schemetatic mechanism of the homolytic ring opening polymerisation of ketene acetal, 2-methylene-1,3-dioxepane (MDO) to form an ester-containing polymer. (B) Preparation of poly(2-methylene-1,3,-dioxepane)-*co*-(acrylic acid).

Our current understanding of formulation polymers relies on non-degradable polymers, typically involving a carbon–carbon backbone. Whilst efforts to synthesise degradable polymer structures by design are ongoing, a method to improve on our current systems is to incorporate breakpoints into traditionally non-degradable polymer. Vinyl and acrylic polymers have been synthesised to incorporate ester functionalities into the polymer backbone *via* the reaction of ketene acetals by ROP (Fig. 4a). Upon homolytic cleavage of the ketene by the initiator or growing radical chain, the ketene π-bond is broken which rearranges to form a carbonyl moiety and in turn produces an ester moiety within the carbon–carbon backbone as well as a reactive radical to propagate the polymerisation with other vinyl monomers. The amount of ketene acetals incorporated can be varied to alter the degradation rate.^71^

Cyclic ketene acetals have been utilised in radical polymerisations, such as atomic transfer radical polymerisations (ATRP), reversible addition-fragmentation chain-transfer (RAFT) polymerisations and nitroxide mediated polymerisation (NMP) mechanisms.^43,44,72^ Furthermore, a variety of different conformations and topologies have been synthesised including homopolymers (*i.e.* polyesters by radical polymerisations)^73^ random co polymerisations as well as di and tri-block co-polymerisations,^71,74^ branched^75^ and star-shaped polymers.^76^ Upon hydrolytic degradation of ketene acetal copolymers, the ester forms acid and diol derivatives of small chain polyacrylates. The resulting oligomeric chains are not susceptible to further hydrolytic degradation. As with polyesters, the degradation rate depends on the neighbouring chain chemistry, the hydrophilicity, and the chain packing. Notably 2-methylene-1,3-dioxepane (MDO) was copolymerised with a *tert*-butyl protected acrylic acid which upon post polymerisation deprotection, gave a degradable poly(acrylic acid)^77^ (Fig. 4b). Degradation of the incorporated ester bonds achieved 66% over 42 hours in pH 8–9 aqueous solution in preliminary tests. In two OECD tests, degradation of the polymer chains as quantified by total oxygen uptake achieved 18–28% over 28 days. The relationship between the number of ester linkage incorporated and degradation rate and any consequential performance alterations are key attributes which would need further investigation before substitution into a formulation. Furthermore, the effect of resultant vinyl oligomers on aquatic ecosystems warrants further investigation to negate any deleterious effects within water systems.

### Biopolymer resources

In redesigning formulation polymers we may take inspiration and build upon the foundations of biopolymers which occur in abundance naturally and make up large portions of agricultural waste. Lignin is a naturally occurring aromatic polymer found abundantly worldwide and a waste product derived from pulping industries.^78^ The exact structural composition varies across plant species but contains both hydrophobic aromatic and ester groups and hydrophilic hydroxyl groups giving it amphiphilic properties. Typical separation of lignin from cellulose and hemicellulose requires high temperatures and harsh reaction conditions which consequently depletes the hydroxyl concentration and hydrophilic potential. Often the functionality of biopolymers require modification to form semi-synthetic polymers which can further optimise solubility, pH and ionic strength. In one recent example, cellulosic-lignin extracted from wood pulp has been extracted and modified under mild conditions with glyoxylic acid.^79^ A carboxylic acid functionality was introduced *via* cyclic ketal formation, which increases the solubility and ease of handling. Furthermore, the authors note that the production does not compete with other lignin processes and instead complements known lignin refinery processes. The resulting polymer, GA-lignin, was able to emulsify mineral oil and water in a pH dependent manner and lowered the surface tension to levels seen for current petrochemical polymers. GA-lignin was then formulated into a hand-cream which demonstrated stability over a 6 month period. Such polymer structures could be useful substitutes for current acid rich branched or crosslinked polymers. Utilisation of a kiloton waste stream with diverse chemical structures presents a unique opportunity. The degradability characteristics and toxicological impacts of a potential aldehyde degradation product remain unknown. The scalability of the system to afford consistent feedstock quality is yet to be investigated and thorough life-cycle analysis is still required.

Polysaccharides are a diverse category of polymer structures which make up a vast majority of structural polymers in the natural world. Polysaccharides exist across plants (cellulose, pectin, starch, xylans), animals (chitosan, heparin), algae (alginates) and microbes (cellulose, dextran) and are characterised by chains of carbohydrate monomers (primarily glucose, fructose and galactose).^47,80^ Sugars are linked by O-glycosidic bonds and can form linear or branched structures. The different types of sugars, the conformation and the molar mass alter the solubility and degradability which presents a variety of potential functionalities.^38,81^

The sustainability of the sourcing and scalability of a polysaccharide biopolymer depends on the type of material and the source from which it derived and future decisions will likely be driven by economic factors. For example, very large-scale utilisation of chitosan from shrimp shells would raise concerns about non-vegan ingredients and the environmental impacts of increased intensive shrimp farming. Even established polymer sources such as starches from root vegetables may impact food production which could lead to increased land used for monoculture farming or inflating prices which would have socioeconomic repercussions. Utilising waste such as the cellulose-rich by-products of wood, paper and agricultural industries presents potentially scalable and sustainable sources.^48^ Citrus fibre has well characterised water-swelling and rheological properties exploited for food and personal care formulations.^42,82^ Cellulose is insoluble in water and is modified to alter the solubility and performance.^38^ Derivatives including dicarboxylic acid nanocellulose,^83,84^ sulfonated nanocellulose^85,86^ as well as cellulose functionalised with acrylic acid^87^ and 2,3-epoxypropyltrimethylammonium chloride^88^ have all been explored as flocculent materials. Numerous modifications to cellulose have been conducted on a small-scale although not all procedures are economical at industrial scale.^89^

While unmodified polysaccharides can be used to deliver thickening and tactile modification properties in home, beauty and personal products, as mentioned previously, derivatisation is commonly used to access a broader range of functionality and performance. The level of derivatisation can be expressed by Degree of Substitution (DS), the average number of hydroxyl groups per repeat unit that are modified during the reaction. DS is used to tune the properties being targeted. Modification of cellulose with chloroacetic acid will form carboxymethyl cellulose or cellulose gum and its neutralised form sodium carboxymethyl cellulose (SCMC). SCMC is an important binder in toothpastes and cleaning aid in laundry products.^90^ The DS must be carefully controlled to achieve the correct solubility and rheology profiles according to the application requirements. Treatment of cellulose in a similar fashion yields other ingredients such as hydroxyethyl cellulose, hydroxypropyl cellulose and hydroxypropyl methylcellulose for rheology modification and can be selected and optimised according to the specifications required.

Starch is another heavily used polysaccharide platform; while hydroxypropyl starch phosphate can be used as a viscosity modifier and emulsifying agent, aluminum starch octenylsuccinate is used in dry shampoo to absorb oils and sweat.

Conditioning agents are commonly used in hair formulations to reduce static, increase smoothness, and improve combing. However, low deposition of these actives is a significant issue in rinse-off formats and polymer-aided deposition is well established for increasing levels retained on the surface of hair. Guar substituted with glycidyl trimethylammonium chloride forms a complex coacervate with surfactant and silicone that will adhere to hair,^91^ with DS of the galactomannan used to tune performance parameters such as complex stability and level of deposition.

Although increasing the DS for polysaccharides often enhances the desired formulation performance, above a certain ceiling, this can come at the expense of reduced biodegradability. It is known that biodegradability of substituted polysaccharides is heavily influenced by the type and number of substituents.^92,93^ While specific enzymes are required to cleave the substituents, steric hindrance around glycosidic linkages in the backbone is also higher.

Polysaccharide degradation occurs by action of a variety of enzymes,^94^ although less than 60% of cellulose is degraded in sludge in wastewater treatment systems.^95,96^ Modification of the properties of a polysaccharide material further alters the degradation potential. Naturally occurring cellulose acetate has different degradation rates depending on the degree of acetylation,^97^ and the biological source should not be presumed as non-toxic.^98^ The abundancy of polysaccharides and their variable properties presents a potentially sustainable source of biopolymers for formulations however the considerable variation in feedstock quality consistency and degradation rates, most notably of modified polymers, in water systems remains a consideration.

### Bio-based synthetic polymers

**Fig. 5:**
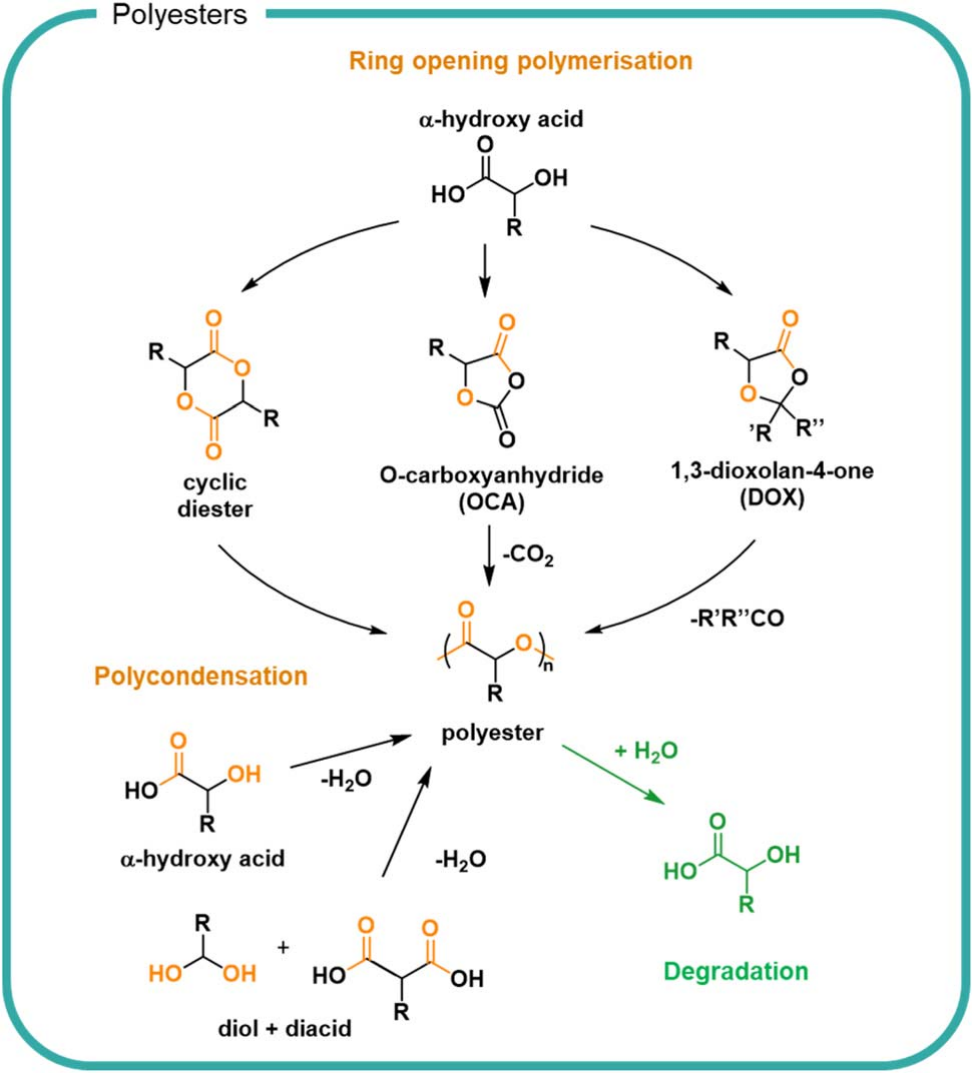
Routes to obtain polyesters from biobased feedstocks including ring opening of cyclic monomers as well as direct polycondensation of small molecules. Degradation of esters can be achieved by hydrolysis to reform the biobased acid or alcohol containing molecules.

Another approach to utilise sustainably derived feedstocks is to chemically process biomass to form small molecules for use as monomers. Ester bonds are ubiquitous in nature and are typically formed by condensation between hydroxyl and carboxylic acid groups (Fig. 5). Naturally occurring polyesters are well known although most polyester materials are made synthetically using natural or synthetic monomers.^99^ The broad functional diversity of polyesters, resulting from the diversity of available monomers, means they find applications as wide-ranging as single-use packaging, textiles and coatings.^100^ Monomer feedstocks for polyesters can be created from biomass to create various polymer structures designed for each application.^101–104^ Conversion is possible by bio-refining biomass; lactic acid can be obtained from corn or biomass can be bacterially fermented to directly form poly(hydroxyalkanoates) (PHAs).^49^ Sustainable design of bio-refining systems allows for a variety of chemical modifications and recycling of incomplete transformation products to minimise losses.^105–107^ For efficiency, synchronised production of multiple compounds for which there is a market should utilise by-products.

Small molecules sourced from biomass^108^ include diacids and diols for stoichiometric polycondensations,^102,109,110^ hydroxyacids for polycondensations,^111–115^ cyclic monomers for ring opening polymerisations,^116–122^ and agents for branching and crosslinking.^111,123^ A notable example utilised across different applications is poly(lactic acid) (PLA), which uses glucose-derived lactic acid as a feedstock.^124^ Lactic acid is a naturally occurring molecule which can be taken up into the Krebs cycle as part of respiration pathways.^125^ Lactic acid can be polymerised directly by polycondensation or cyclised for ring opening polymerisation, as shown in Fig. 5. The different polymerisation methods allow for different control of reaction, molar mass, and dispersity of product. Polyesters have found most use in durable materials for clothing and packaging owing to the hydrophobic nature of many polyesters which has limited both its variety of applications and degradation.

The ester bond is susceptible to both enzymatic degradation by a variety of esterases, lipases and cutinases as well as hydrolytic degradation under mild conditions. In both cases, an acid and alcohol are formed from each ester. The rates of which are determined by the specific enzyme^126^ or surrounding medium as well as the chemical structure of the polymer.^67,100,127,128^ A key determinant of hydrolysis is the hydrophilicity of the polymer which itself is determined in part by the crystallinity and packing of the polymer chains, where closer packing and increased crystallinity slow the degradation rate. Aromatic and highly hydrophobic bio-based polyesters such as poly(ethylene 2,5-furanoate) (PEF) are not susceptible to hydrolysis although the bonds can be cleaved by chemical, mechanical and specialist enzymatic means.^129^ The ordered structure of the PEF and the pi–pi stacking of the aromatic rings allow for close packing of the chains and thus a poorly soluble materials with strong resistance to hydrolysis. Aliphatic polyesters with regular repeat units such as PLA are also hydrophobic but can undergo degradation by hydrolysis, albeit with rates which are typically slow (<10% after 116 weeks in deuterated water at 25 °C).^130^ Decreasing the crystallinity of polyester PLA can be achieved by copolymerisation with glycolic acid monomers, to form poly(lactic-glycolic acid) (PLGA) copolymers which decreases the glass transition temperature and increases the degradation rate to 65–75% after 116 weeks in 25 °C deuterated water.^131^ As a result PLGA has been shown useful for pharmaceutical formulations.^132^ The inclusion of hydrophilic polymer functional groups such as alcohols, sulfonates, quaternary ammoniums and carboxylic acid groups have been investigated to increase solubility.^133^ Highly soluble polyesters have been synthesised by the polycondensation of malic acid, leading to branched acid rich polymers with a molar mass up to 3000 g mol^−1^.^134^ Owing to the increased solubility, rapid degradation of ester bonds to reform the malic acid monomers was observed in water at varying pH and showed complete degradation in 2 weeks in pH 7 solution.^114^ Copolymerisation of malic acid with lactic acid (74%) was shown to decrease the rate of degradation to 50% mass loss over 10 weeks in pH 7.4 buffer.^135^ By increasing the lactic acid content with respect to the malic acid the rate could be slowed further. Choice of monomer therefore plays a significant role in the degradability of the resulting polymer.

**Fig. 6:**
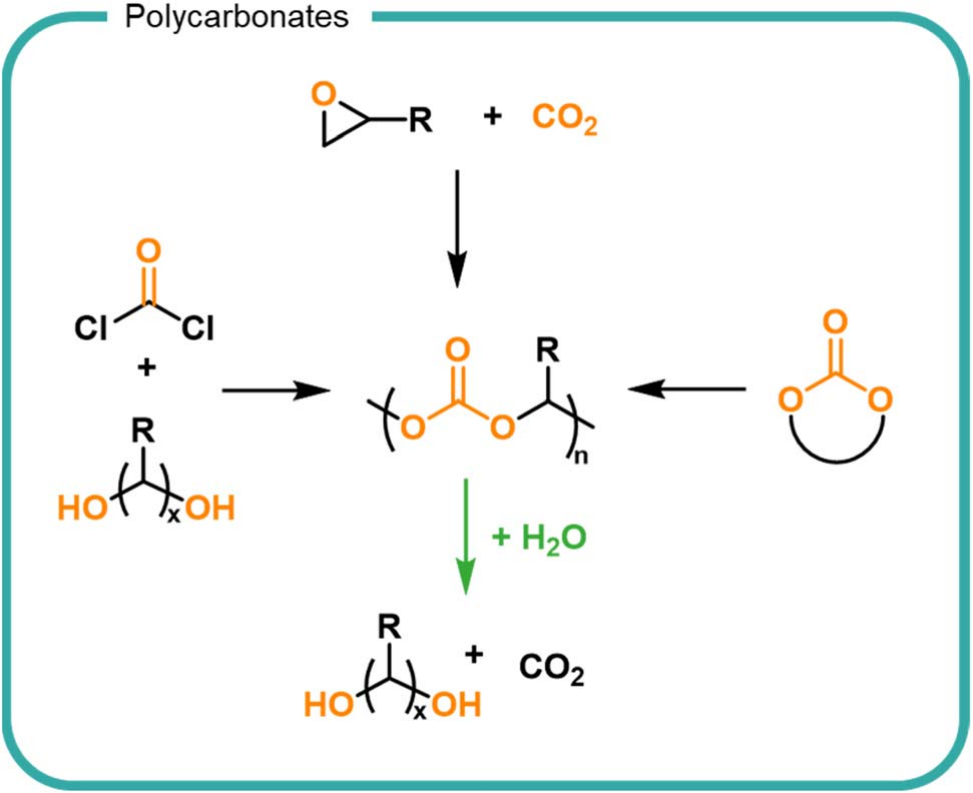
The synthesis and degradation routes of polycarbonates including ring opening of cyclic esters and epoxides as well as the step-growth addition of diols and phosgene. Upon degradation, carbon dioxide is released forming hydroxyl species.

A synthetic structural relative of polyesters are polycarbonates which are characterised by the carbonate linkages in the backbone (Fig. 6). Polycarbonates are not naturally occurring but are attractive owing to their utilisation of CO_2_ embedded into the polymer backbone. Traditionally carbonate bonds have been synthesised by using phosgene as a carbonyl source *via* a polycondensation mechanism with diols. More recently, the development of highly specific catalysts have led to CO_2_ incorporation into epoxides as well as the cyclisation of diols *via* the incorporation of CO_2_, both of which form cyclic carbonates which serve as monomers for the formation of polycarbonates by ROP.^136–138^ Carbonates undergo hydrolysis to release CO_2_ leaving hydroxyl functionalised small molecules (Fig. 6).^139,140^ Degradation rates are slower than their ester counterparts owing to the increased resonance of the additional oxygen–carbon bond. The release of carbon dioxide from the system drives degradation to completion. The ability to utilise CO_2_ as a feedstock for future materials provides an opportunity for carbon sequestration into useful material products. Degradation of polycarbonates must be controlled to prevent the premature release of carbon dioxide in a formulation.

Current technologies to obtain polycarbonates *via* a sustainable route are limited for use in FMCGs by the high cost of production, lending them to higher value applications.^138–141^ Polyesters and polycarbonates offer an opportunity to create finely tuned polymers from biomass which contain multiple cleavage points to return the structure back to small molecules. As with all biomass utilisation, the sources should be considered to ensure a sustainable production which is both scalable and economically viable. Choice in monomers and synthetic strategy is driven by the needs (*i.e.*, the performance and degradation) of the polymer. Degradation of esters and carbonate groups presents an opportunity for hydrolytic degradation without the reliance on enzyme-rich environments such as wastewater treatment plants. However, such chemistries should be utilised with care to prevent premature degradation and thus instability within a formulation. Economically, bio-based feedstocks are currently outpriced by fossil-derived alternatives, however opportunities are predicted as consumer pressure and government legislations are driving demand for bio-derived products.^49,142^

Polyamides show great potential for formulation polymers owing to their variable side chains, stability against hydrolytic degradation and their susceptibility to a variety of peptidase enzymes as a mechanism for degradation. Many amino acids can be formed from biomass by bacterial fermentation which presents a route to form a desired product at scale with high purity and often at low temperatures.^143^

**Fig. 7:**
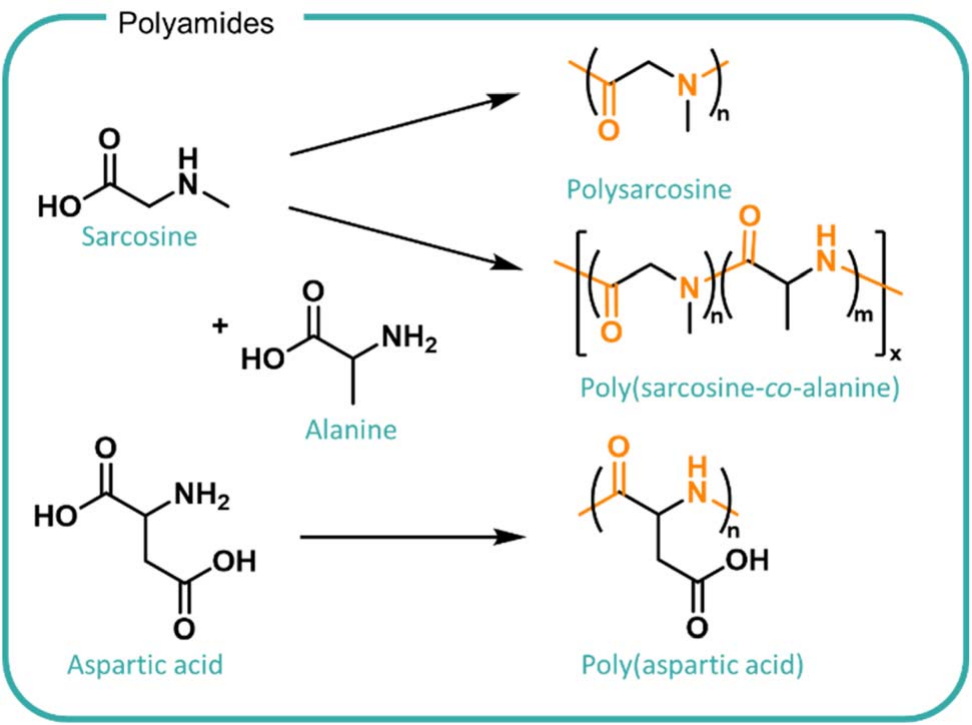
Structures of polyamides derived from amino acids, sarcosine, alanine and aspartic acid. (Top) Sarcosine can be polymerised to form polysarcosine and copolymerised with alanine to form poly(sarcosine-alanine). The inclusion of alanine comonomers promotes enzymatic degradation by porcine pancreatic elastase.^147^ (Bottom) Poly(aspartic acid) is synthesised from aspartic acid which includes an amide backbone and carboxylic acid functional groups which increases the solubility and acts as branching points.

In principle, peptide bonds can be cleaved enzymatically although the nature and sequence of monomers as well as the conformation and hydrophilicity of the polyamide in solution can modulate enzyme activity so far as completely removing its degradability. Enzymes are highly selective for specific substrates which is determined by the structure of the enzyme active site. The chemical bond in question and the adjacent polymer chains needs to be able to access the enzyme active site in the right conformation in order for binding and subsequent cleavage to occur. The specificity of different enzymes found in different geographic and situational locations should be considered when designing polymers for different applications. For example, polysarcosine has been investigated as a biodegradable alternative to PEG (Fig. 7). Despite being comprised of a naturally occurring derivative of amino acids and possessing good water solubility, it demonstrates limited enzymatic biodegradability.^144,145^ Incorporation of alanine monomers to form the poly(sarcosine-alanine) copolymer facilitated degradation by the use of porcine pancreatic elastase to over 50 days at pH 8 at 37 °C.^146^ Whilst effective, the requirement for a collected waste stream within a closed system presents challenges with scaling to national systems including availability, cost, efficiency and cross-contamination risks. Furthermore, when disposed of in increasingly diverse wastewater systems where enzyme concentration or effective activity is poorly understood, the polymer may not degrade, posing a risk to ecosystems.

Poly(aspartic acid) (PASP, shown in Fig. 7) also shows promise for the utilisation of bio-derived amino acids as a water swellable formulation polymer and is investigated as a biodegradable analogue to polycarboxylates.^8^ PASP was synthesised by co-extrusion, ring-opening polymerisation and polycondensation methods leading to different sizes and architectures.^147^ The work demonstrated effects of structure on biodegradability in activated sludge tests conducted at the lab scale. Smaller linear polymers exhibited higher conversions of total organic content (up to 95%) whereas the larger, branched structures were only partially degraded (45%) after 28 days. Modifications to incorporate succinimide groups onto the chain ends led to a further decrease in biodegradation. The formation of non-degradable products suggests potential polymer adsorbance onto the sludge which may accumulate in the environment.^8^ The PASP degradation is designed to utilise existing waste treatments and reports relevant biodegradation outcomes in activated sludge however, information on the fate and impact on the release of polyamides directly into water streams remains unknown.

### Self-assembled polymer structures

**Fig. 8:**
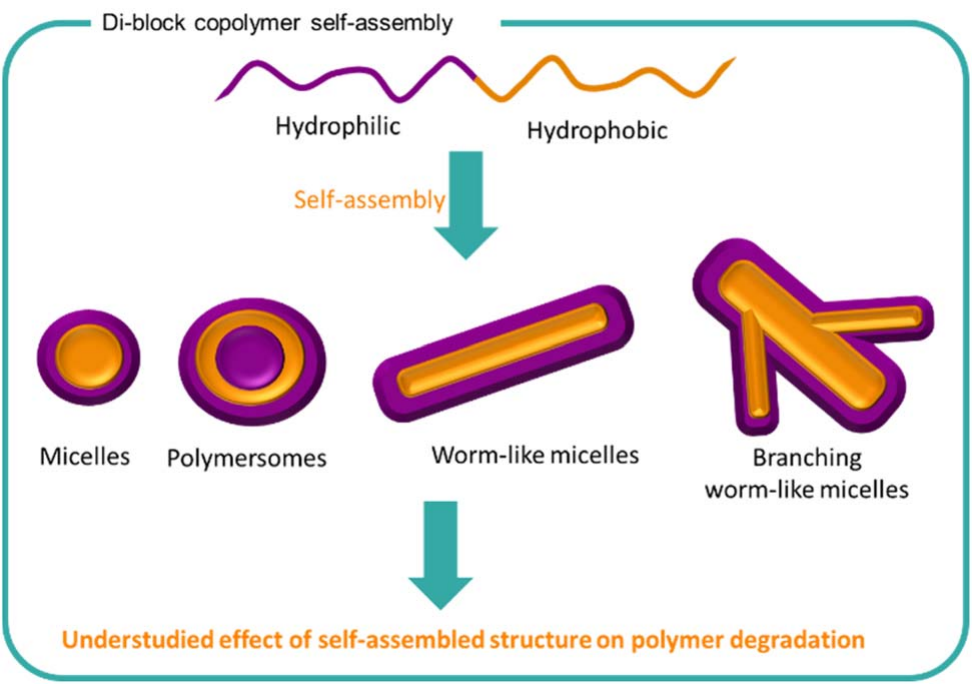
The self-assembly of amphiphilic block copolymers into a variety of structures is well known. The effect of polymer self-assembly on the degradation of the polymer chains is less understood.

Self-assembled structures are used widely in the natural world as well as in a variety of synthetic formulations.^89,148–151^ From surfactants to milk to drug delivery systems, molecules and polymers arrange themselves into structures to lower the energy of the system. The process of self-assembly is driven by areas of poor solubility within a medium which forces the clustering of such structures together. Typically, amphiphilic molecules consist of both charged groups such as sulfates or quaternary ammoniums with hydrophobic regions such as alkyl chains which causes duality in behaviour. Amphiphilic polymers such as block polymers are commonly utilised for creating different self-assembly systems including micelles, vesicles, polymersomes, bilayers, tubules, gels and worm-like morphologies (Fig. 8).^152^ The resulting morphology is highly dependent on the nature of the polymer and the medium with shape, size, solubility, and conformation being key factors. The arrangement of polymers chains into large nano to macrostructures changes the behaviour of the formulation including stabilisation of other ingredients, turbidity, viscosity and potential performance effects.

**Fig. 9:**
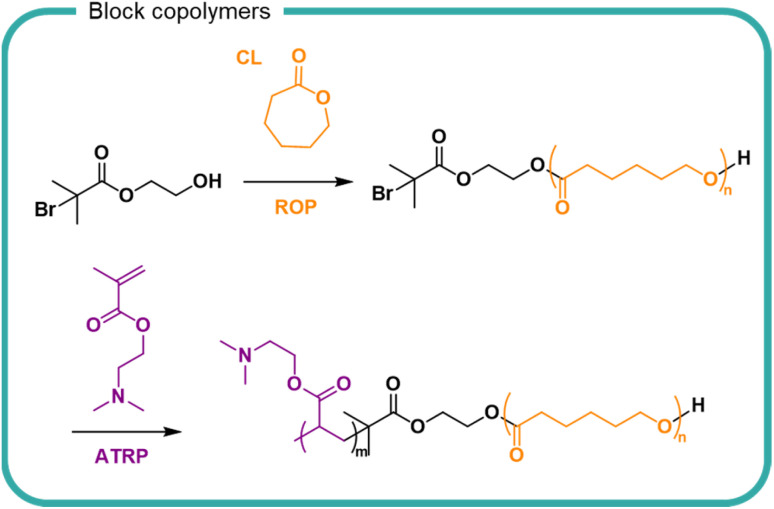
The synthesis of poly(caprolactone)-*block*-poly(*N*,*N*-dimethyleminoethylmethacrylate) (PCL-PDMAEMA) by the ring opening polymerisation (ROP) of caprolactone (CL) followed by the radical polymerisation of *N*,*N*-dimethylaminoethyl methacrylate by anionic chain transfer radical polymerisation (ATRP).^158^

Stability of self-assembled structures and their encapsulated cargo (*e.g.* pharmaceuticals, proteins, insoluble ingredients) are well documented in the literature with considerable attention in the biomedical field. For hydrolytically susceptible cargoes, such as lactone-containing compounds, encapsulation into self assembling block copolymers (either by conjugation or electrostatic interactions), can increase hydrolytic stability and solubility.^153,154^ In recent years there has been greater interest in the use of degradable polymers for self-assembly structures, including polyesters such as PLGA, PLA and polycaprolactone (PCL)^155–157^ The degradation of PCL in a poly(*N*,*N*′-dimethylaminoethyl methacrylate) block copolymer PCL-PDMAEMA micelles has been studied (Fig. 9).^158^ Degradation of PCL in the PCL-PDMAEMA micelle increased compared to homopolymer degradation by 30% in buffered solutions over the 6 week study. Conversely, for enzymatic degradation, the rate of degradation decreased compared to PCL homopolymer which further decreased as a function of increasing PDMAEMA length. In the block copolymer form, the solubility of PCL and its surface area are increased, hence an increase in degradation comparatively to its insoluble homopolymer counterpart. The degradation of a block copolymer of poly(malic acid-*co*-lactic acid) (PMLA) and PEG, in which the anionic PMLA is ordinarily hydrophilic and rapidly degradable, was slowed by self-assembly into nanoparticles in the presence of cationic doxorubicin.^159^ PMLA and doxorubicin formed a complex which became hydrophobic and induced the self-assembly of nanoparticles with PEG chains surrounding the hydrophobic core. Doxorubicin release from the nanoparticles was studied over a 70 hour study period, showing that both low pH and high sodium chloride concentration destabilised the ionic core of the nanoparticle. Degradation of the polymer was not studied, either as a homopolymer or block copolymer.

Theoretical studies show that the inclusion of degradable polymer segments within the core or corona segments of block copolymer micelles demonstrate different stability profiles of the structure.^160^ Core degradation results in destabilisation leading to smaller micelles whilst corona degradation results in the formation of larger micelles. Rather than destruction of self-assembled structures upon polymer degradation, the hydrolytic degradation of a PLA-PEG-PLA triblock copolymer induced reorganisation of self-assembled structure leading to gel formation.^161^ The triblock polymers of differing molar masses, initially formed micelles in water which upon hydrolytic degradation of the PLA chains formed hydrophobic channels between the micelles, extending the network and resulting in a gel material. Furthermore, enzyme-mediated degradation of di-block and tri-block polymers has shown to induce structural changes from micelles to cargo-encapsulating gel networks and subsequent polymer degradation.^162^ Changing the ratio of the hydrophilic and hydrophobic blocks could control the rate of structural transitions facilitating a degree of programmable stability to a self-assembled system. The changing structures and physical properties of the formulation upon degradation could be exploited in liquid formulations. Whilst the morphology was investigated both theoretically and experimentally for the PLA-PEG-PLA triblock polymer, an extension of this study to understand similar effects of different polymers would provide valuable insight.

The effect of polymer structure on the rate and type of self-assembled morphology is well documented.^157,163,164^ Extrapolating from this, the type of self-assembled structure will alter the stability of the system and polymer chains. To our knowledge, and perhaps a consequence of the complexity of the systems involved, there remains a gap in the knowledge in understanding and predicting the relationship between self-assembled structures and polymer degradation. Understanding the degradation of the polymer within different self-assembled morphologies remains an interesting and important area for polymer chemists to investigate for liquid formulations.

## Future perspective

Sustainability is complex, especially for formulation polymers, as many factors must be considered in harmony to design and develop robust sustainable systems. Bio-based feedstocks are potentially available to form numerous polymeric structures, but only with judicious use of our available resources can we design robustly sustainable chemical systems. As we begin to phase out non-degradable and fossil-derived polymers, we must ensure that their replacements are holistically sustainable by considering the wider implications of resource utilisation; life cycle analysis from cradle to grave, economic and environmental consequences. Furthermore, understanding the variety of end destinations of formulations is pivotal in building truly degradable polymers and avoiding the release of non-degradable polymers into the biosphere.

This poses a major challenge for the next 5–10 years of research in future-proofing FMCG formulation polymers. Increased transparency and collaboration, especially in traditionally closed sectors, can embed change more rapidly. Cross-sector collaboration can also help reshape regulation. Current OECD standards on biodegradation are not fit for purpose for new polymer development, as they are onerous and can be expensive, especially considering the diversity of environments of eventual release. We must ensure that systems are designed to avoid environmental impact regardless of where and how these formulation polymers are released. This is best enabled by academics, corporations and regulators working with each other to redevelop these important standards. Understanding and predicting environmental degradation as a physical, chemical, and biological process is key to avoiding unintended consequences. This interdisciplinary approach underlines the need to ensure safety at every stage of degradation – for polymers this hinges on the ecotoxicity of polymeric, oligomeric, and monomeric fragments which exist on the biodegradation pathway.

Similar efforts to evaluate the sustainability of new formulation polymers will also aid in product development. While cradle to gate life cycle assessments are now commonplace in chemical process development, extending these calculations to full environmental impact assessments and to true end-of-life scenarios is important. Cross-sector collaboration to help define boundaries for life cycle assessments will be key to quantifying impact. Mapping LCA data onto performance and economic sustainability should be used to help triage development options as technologies scale.

Transitioning FMCGs to a suite of future polymers fit for the circular economy will help to deliver superior consumer products which are more sustainable than today. Making this transition will require concerted effort across the chemical, chemical-using industries and their research partners developing the feedstocks, monomers and polymers needed for high performing, affordable consumer goods. The key challenges to be overcome in this transition have been outlined herein, and whilst they are significant we hope that a concerted effort from researchers across academia and industry will enable this critical sectoral transition to more sustainable polymers, in a way that is affordable for consumers.

## Author contributions

Christina Picken: conceptualization, writing – original draft, editing. Orla Buensoz: conceptualization, writing – original draft, editing. Paul Price: funding acquisition, writing – review & editing. Christopher Fidge: funding acquisition, writing – review & editing. Laurie Points: writing – review & editing Michael P. Shaver: conceptualization, funding acquisition, supervision, writing – review & editing.

## Conflicts of interest

Unilever provided sponsorship for this project as part of their efforts to improve product and process sustainability.

## Supplementary Material
